# Comparison of propofol-nalbuphine and propofol-fentanyl sedation for patients undergoing endoscopic retrograde cholangiopancreatography: a double-blind, randomized controlled trial

**DOI:** 10.1186/s12871-022-01578-9

**Published:** 2022-02-16

**Authors:** Peiqi Wang, Yan Chen, Ying Guo, Jiangbei Cao, Hong Wang, Weidong Mi, Longhe Xu

**Affiliations:** 1 Department of Anaesthesiology, The First Medical Center, Chinese PLA General Hospital, Beijing, 100853 China; 2 Department of Anaesthesiology, The Third Medical Center, Chinese PLA General Hospital, Beijing, 100853 China

**Keywords:** ERCP, Nalbuphine, Fentanyl, Sedation, Adverse effects

## Abstract

**Background:**

Endoscopic retrograde cholangiopancreatography (ERCP) has been increasingly used to treat patients with biliary/pancreatic duct obstruction or stricture outside the operating room. Effective and safe sedation techniques are needed because of painful stimuli and the long duration of the ERCP procedure.Nalbuphine has been shown to cause less respiratory depression during sedation than similar cases without nalbuphine. This study compared the effects of propofol-nalbuphine (PN) and propofol-fentanyl (PF) sedation in patients undergoing ERCP.

**Methods:**

Four hundred patients scheduled for ERCP procedures were divided into two groups: the PF group (receiving PF sedation,*n* = 199) and the PN group (receiving PN sedation,*n* = 201). Vital signs, adverse events during surgery, patient movement scores, pain scores, and adverse events one day post-ERCP were recorded.

**Results:**

Stable haemodynamics were observed in both groups.Compared to the PF group, the PN group showed significantly decreased respiratory depression (*P* < 0.0001) and surgical interruptions (*P* = 0.048).Nalbuphine decreased patient movement by reducing pain from ERCP.

**Conclusion:**

Nalbuphine, instead of fentanyl, precipitated less respiratory depression while permitting adequate/equivalent sedation for ERCP and therefore provides more efficient and safer sedation.

Trial registration

ChiCTR, ChiCTR1800016018, Registered 7 May 2018, http://www.chictr.org.cn/showproj.aspx?proj=27085

## Background

Endoscopic retrograde cholangiopancreatography(ERCP) is performed by gastroenterologists or surgeons to investigate abnormalities of the common bile duct, pancreatic ducts, and ampulla. It can also be used to perform certain therapeutic interventions [1]. Although the decrease in diagnostic ERCP was largely precipitated by magnetic resonance cholangiopancreatography (MRCP) and endoscopic ultrasound (EUS) [2, 3],ERCP has been increasingly used to treat biliary/pancreatic duct blockage or narrowing by stones,tumours,or inflammation outside the operating room [4,5,6]. The procedure lasts from 30 to 60 min, and the patients need to be in the prone or semiprone position [7]. Patients usually cannot tolerate the procedure because of pain, the uncomfortable position,fear and nausea without adequate sedation [8].Therefore, ERCP is generally performed under moderate to deep sedation with adequate analgesia to ensure the success of the procedure and improve patient comfort [9].If used as the only anaesthetic agent,an increased dose of propofol may cause undesirable side effects while lacking adequate analgesic effects to inhibit visceral traction pain [10]. Compared to the administration of propofol alone,the application of propofol with fentanyl for ERCP can reduce the total dose of propofol, decrease the pain level, increase practitioner satisfaction, and provide haemodynamic stability but is more likely to induce respiratory depression, muscle stiffness and airway obstruction [11, 12].

Hypoxia is a common occurrence during upper GI endoscopy under sedation [13, 14], and prolonged hypoxia is the most common cause of cardiac arrhythmia and coronary ischaemia [13]. Avoiding respiratory depression is helpful to reducethe incidence of hypoxia and has been recommended by the American Society of Anesthesiologists and the American Society for Gastrointestinal Endoscopy [15].Nalbuphine is a mixed agonist–antagonist opioid with a duration of action of approximately 3–6 h and causes less respiratory depression than fentanyl [16, 17]. It has also been used to treat pain over the past 40 years [18]. Considering the unique pharmacology in pain management [19], nalbuphine is possibly superior to fentanyl in ERCP due to the lower respiratory depression and adequate analgesia provided by nalbuphine.

In this prospective, double-blind, randomized controlled trial, we compared the analgesic efficacy and safety of propofol combined with nalbuphine or fentanyl in patients undergoing ERCP.We hypothesized that patients sedated with nalbuphine and propofol would have a lower rate of hypoxia during ERCP procedures than patients sedated with fentanyl and propofol.

## Methods

### Study design

This prospective, double-blind, randomized controlled clinical trial was approved by the ethics committee of Chinese PLA General Hospital (S2017-075–02) and was registered in the Chinese Clinical Trial Registry (ChiCTR1800016018,07/05/2018)before its initiation in May 2018. Written informed consent was obtained from all patients.All investigators, including nurses and anaesthetists, received standardized training.

### Patient recruitment and exclusion

The patients included in this study were those who were scheduled for elective ERCP, aged 18 to 79 years, had a BMI of 18.5 ~ 30 kg.m^−2^ and were classified as ASA I-III. The exclusion criteria were as follows: (i) clear diagnosis of heart disease (heart failure,angina, myocardial infarction, arrhythmia, etc.); (ii) clear diagnosis of pulmonary disease (asthma, chronic obstructive pulmonary disease, pulmonary embolism, pulmonary oedema, or lung cancer); (iii) central nervous system abnormality; (iv) allergy to the study drugs; (v)pregnancy; and (vi)anticipated difficult airway.

### Grouping design and study procedure

The patients were randomly divided into 2 groups, the PN group(propofol and nalbuphine) and the PF group (propofol and fentanyl),by using a computer-generated randomization table. All of the patients were admitted to the hospital at least 1 day before ERCP.

The procedure was performed in the endoscopy suite by 1 of the 3 gastroenterologists in the institution, who had the experience of performing at least 500 ERCP procedures each. An experienced anaesthesiologist administered the drugs, and an anaesthesia resident collected the clinical data. We prefilled 5-ml syringes with either 4 ml of liquid fentanyl (50 μg ml^−1^) or 4 ml nalbuphine (5 mg ml^−1^) and labelled them with randomized numbers. In this way, all patients, the anaesthesiologist, the anaesthesia resident and the gastroenterologists were blinded to the group information.

All patients were placed in prone position. They all received midazolam and propofol for sedation and were then monitored for sedation depth using the bispectral index (BIS). Midazolam (0.01–0.02 mg kg^−1^) was initially administered. Patients in the PN group received 0.1–0.2 mgkg^−1^nalbuphine and 1–2 mgkg^−1^ propofol intravenous injection over 30s followed by an infusion of propofol at 0.05 to 0.1 mg (kg min)^−1^, and patients in the PF group received 1–2 µgkg^−1^ fentanyl and 1–2 mgkg^−1^ propofol injection over 30s followed by an infusion of propofol at 0.05 to 0.1 mg (kg min)^−1^.The BIS index was used to assess the level of sedation, and a value of 40–60 was targeted for the procedure [20]. In cases of an index > 60 or sudden patient movement, propofol 20–30mg + nalbuphine 5mg or propofol 20–30mg + fentanyl 50µg were given in an intravenous bolus to the assigned groups as rescue drugs. The procedure was allowed to start at the discretion of the attending anaesthesiologist.

Baseline vital signs were recorded immediately before the procedure. Oxygen was delivered by nasalprongs at 4Lmin^−1^ or by an endoscopic facial mask at 4 Lmin^−1^, and a balanced salt solution at 4mL (kghr)^−1^ was givenduring the procedure. All patients received BIS (BIS VISTA™ monitoring system, Aspect Medical Systems Inc., Norwood, MA, USA) at 1-min intervals for the first 3min after induction and every 3min thereafter. Heart rate (HR), noninvasive mean blood pressure (NBP),respiratory rate (RR), and oxygen saturation (SpO_2_) were documented every 5min. The situation regarding surgical interruptions was recorded. A modified Aldrete score of 9 was considered recovery, and the patient was transported back to the ward after meeting discharge criteria. The score of patient movement was marked during the operation as follows: no movement (10 points); slight movement, occasionally(8 points); slight movement, frequently(6 points);severe movement only with the arms and legs (4 points); severe movement with the body and head (2 points).The endoscopist and attending anaesthesiologist assessed the process of the procedure at the end and rated it as (I) satisfactory,(II)indeterminate, and(III) not satisfactory. All of the patients were interviewed by the patient sedation satisfaction assessment tool (PSSI) regarding their experience 1 day after the operation.Adverse effects such as nausea, vomiting, pruritus, polypnea and dyspnoea, pain score(visual analogue score,VAS),etc. were noted.The total dose of the study drug and propofol in each group were calculated.

Hypoxia-related adverse events were described as follows: subclinical respiratory depression (90 ≤ SPO2 < 95% for > 10 s)and hypoxia (SpO2 < 90% for > 10 s). There were two degrees of hypoxia: mild risk (75 ≤ SPO2 < 89%)and severe risk (SPO2 < 75%). It was corrected using the following protocols: (I) opening the airway with the jaw-thrust manoeuvre; (II) placing the nasopharyngeal airway; (III) turning over to the supine position; and (IV) tracheal intubation for mechanical ventilation.The endoscope was removed if hypoxemia could not be corrected. The attending anaesthesiologist provided immediate airway support, and the procedure was resumed or abandoned at the discretion of the anaesthesiologist. Hypotension and bradycardia were treated with intravenous ephedrine 0.1 mgkg^−1^ and atropine 10 µgkg^−1^,respectively.

### Randomization and sample size estimations

A computer-generated randomizationtable was used for the study centre. The randomization sequence was generated by a research assistant who was independent of the study and did not have contact with the study participants.Randomization was performed using opaque sealed envelopes before the induction of anaesthesia.

In this study, the sample size was estimated based on the incidence of hypoxia (SPO_2_ < 90% for > 10 s) as the main evaluation index. According to the literature, the incidence of SPO_2_ less than 90% and over 10s is 42.8% in ERCP when using fentanyl and propofol for sedation [10, 11]. It was assumed that the incidence of hypoxemia in the PN group was comparable to that in the PF group.Assuming an α value of 0.025, an a β value of 0.2 and a δ value of 0.15, we needed a sample size of 171 for each group according to PASS 11 (NCSS, LLC., Kaysville, UT, USA) software. Allowing for a dropout rate of 10%, we calculated that a minimum of 380 cases would need to be enrolled. Considering potential loss to follow-up, we increased the sample size of each group to 200 patients.

### Statistical analysis

The data are shown as the mean ± SD or percentage as appropriate. Thet test was used to compare the two groups, and if the data distribution was skewed, the Wilcoxon test was used.The χ^2^ test and Fisher’s exact test were used to compare the incidence of adverse events between the two groups. A p value < 0.05 was considered statistically significant.All statistical analyses were performed using SPSS version 16(SPSS Inc., Chicago, IL, USA).

## Results

This study recruited 400 patients scheduled for ERCP from May 2018 to June 2019 at our centre.The patients were randomized to two groups.The PN group included 201 patients, and the PF group had 199 patients. The patients’ characteristics(age, sex, BMI, ASA classification, and Mallampati scores)and procedure details(surgical time and types of the procedure)are compared in Table 1 (*p* > 0.05). Most of the procedures were performed within one hour.Choledocholithiasis was the most common indication for ERCP.

**Table 1:** Patient characteristics and procedure details

	GroupPF(*n* = 199)	GroupPN(*n* = 201)
Age(yr)	58.09 ± 13.51	57.91 ± 13.42
Age range(yr)	21–79	24–79
Age grouping(n,18–64/65-79 yr)	132/67	133/68
Gender(male/female)	132/67	124/77
BMI(kgm^−2^)	22.68 ± 3.63	22.86 ± 3.15
Mallampati Class(n,I/II/III)	14/183/2	13/185/3
ASA grading(n,I/II/III)	22/131/46	19/132/50
Duration of anaesthesia(min)	44.91 ± 28.77	43.73 ± 24.76
Duration of operation(min)	37.43 ± 27.96	36.62 ± 24.15
ERCP indication(n,%)		
Choledocholithiasis	105(52.76%)	93(46.28%)
Gall bladder carcinoma	16(8.04%)	15(7.47%)
Biliary strictures	16(8.04%)	22(10.95%)
Pancreatic pathology	24(12.06%)	33(16.39%)
Pancreatic carcinoma	20(10.05%)	23(11.44%)
Others	18(9.05%)	15(7.47%)

There were no significant differences in the haemodynamic features of the patients (HR, MAP, RR, and SpO2) between the two groups(*p* > 0.05),although the PF group showed greater fluctuation than the PN group did in HR, MAP and SpO2.

Respiratory depression was the primary outcome, and surgical interruptions were the secondary outcome in the study.Adverse events such as respiratory depression and interruptions during the procedure are listed in Table 2.The PN group had a significant decrease in the total percentage of respiratory depression compared with that in the PF group (*p* < 0.0001,95% CI:1.04–3.93). Fourteen (14/199,7.04%,*p* = 0.03,95% CI: 0.86–1.88) patients had hypoxia, and 5 (5/199,2.51%,*p* = 0.048,95% CI: 1.17–2.45) patients developed severe hypoxia in the PF group, while there were only 6 (6/201,2.99%) patients with hypoxia and 1(1/201,0.50%) patient with severe hypoxia in the PN group.Respiratory depression was not associated with age, as patients aged 18–64 yr (*p* < 0.0001,95% CI: 1.04–1.94) and 65–79 yr (*p* < 0.0001,95% CI: 0.87–2.07) had more respiratory depression in the PF group than in the PN group.

**Table 2:** Adverse events and interruptions during the procedure

	GroupPF(*N* = 199)	GroupPN(*N* = 201)	*p* value	95% CI	Odds ratio
Respiratory depression(n)% ^a^	24 (12.06%)	12 (5.97%)	< 0.0001	1.04–3.93	2.16
subclinical respiratory ^a^ depression(n)% ^a^	10 (5.03%)	6 (2.99%)	0.14	0.86–1.88	1.72
hypoxia(n)% ^a^	14 (7.04%)	6 (2.99%)	0.03	1.06–1.95	2.46
minor risk hypoxia(n)% ^a^	9(4.52%)	5(2.49%)	0.13	0.87–1.96	1.86
severe risk hypoxia(n)% ^a^	5(2.51%)	1(0.50%)	0.048	1.17–2.45	1.69
18-65y Age-related Respiratory depression(n)% ^a^	15(11.36%)	7(5.26%)	< 0.0001	1.04–1.94	2.31
66-80y Age-related Respiratory depression(n)% ^a^	9(13.43%)	5(7.35%)	< 0.0001	0.87–2.07	1.96
Correction of hypoxia(n)% ^a^	24 (12.06%)	12 (5.97%)	< 0.0001	1.04–3.93	2.16
Method I(n)% ^a^	12 (6.03%)	8 (3.98%)	0.24	0.84–1.77	1.55
Methods I + II(n)% ^a^	4 (2.01%)	1 (0.50%)	0.09	1.03–2.54	1.62
Methods I + II + III(n)% ^a^	6 (3.02%)	3 (1.49%)	0.15	0.84–2.17	2.05
Methods I + II + III + IV(n)% ^a^	2 (1.01%)	0 (0.00%)	0.07	1.83–2.23	2.02
Surgical interruptions(n)% ^a^	45 (22.61%)	30 (14.93%)	0.02	1.02–1.57	1.27
The score for patient movement(points)^b^	9.61 ± 1.01	9.59 ± 1.07	0.37	-0.23–0.25	–––

Values are presented as the frequency (%),Chi-squared test^a^.Values are presented as the mean ± SD, one-sample t test ^b^. Methods: (I) opening the airway with the jaw-thrust manoeuvre; (II) placing the nasopharyngeal airway; (III) turningthe patients over to the supine position; and (IV) tracheal intubation for mechanical ventilation

In the PN group, 12(12/201, 5.97%) patients received a combined method of correcting respiratory depression, which was significantly lower (*p* < 0.0001,95% CI: 1.04–3.93) than that in the PF group (24/199, 12.06%).There were 8 patients who needed assisted ventilation with jaw thrusts in the PN group and 12 such patients in the PFgroup.One patient in the PN group require da jaw thrust and a nasopharyngeal airway versus 4 patients in the PF group. There were 3 patients in the PN group and 6 patients in the PF group who were turned to the supine position and received assisted ventilation with two methods.Two patients needed tracheal intubation for mechanical ventilation in the PF group, while no patient had tracheal intubation in the PN group.There were no differences in the rescue methods between the two groups.

In the PFgroup, 45 (45/199, 22.61%) cases were interrupted during the procedure by patient movement or desaturation (2 patients underwent tracheal intubation), while 30, 30/201, 14.93%) cases were interrupted in the PN group (*p* = 0.02,95% CI: 1.02–1.57). There were no differences in the score of patient movement between the two groups.

The total doses of drugs, including midazolam,propofol, fentanyl, nalbuphine, ephedrine, and atropine, are shown in Table 3(p > 0.05).

**Table 3:** Total dose of drugs given during ERCP

Total dose of drugs	GroupPF(*n* = 199)	GroupPN(*n* = 201)
Midazolam(mg) ^b^	1.09 ± 0.26	1.09 ± 0.25
Propofol(mg) ^b^	229.4 ± 139.0	234.6 ± 118.2
Study drug(ml) ^b^	2.21 ± 0.62	2.14 ± 0.53
Intravenous ephedrine(n,%) ^a^	7 (3.52%)	7 (3.48%)
Atropine(n,%) ^a^	15 (7.54%)	20 (9.95%)

Values are presented as the frequency (%),Chi-squared test^a^.Values are presented as the mean ± SD, one-sample t test ^b^.Study drug: fentanyl (50 μg/ml) or nalbuphine (5 mg/ml)

The endoscopists rated more cases as (III) not satisfactoryin the PF group (Table 4). Eight patients in the PF group and 11 in the PN group were rated as (II) indeterminate. Additionally, anaesthesiologists rated fewer cases as (III) not satisfactory and (II) indeterminate in the PN group. There were no differences in the satisfaction scores of either endoscopistsor anaesthesiologists between the two groups(*p* > 0.05). The satisfaction scores of the patients were similar in both groups (*p* > 0.05). They were discharged approximately seven days after surgery, and no significant differences were observed in hospitalization days between the two groups (*p* > 0.05).

**Table 4:** Satisfaction score and hospitalization days

	GroupPF(*n* = 199)	GroupPN(*n* = 201)
Endoscopist score(n,I/II/III) ^a^	188/8/3	189/11/1
Anaesthesiologistscore(n,I/II/III) ^a^	178/19/2	185/15/1
PSSI score(points) ^b^	21.10 ± 3.82	21.01 ± 4.08
Hospitalization days(d) ^b^	7.93 ± 9.80	7.42 ± 7.02

Values are presented as the frequency (%),Chi-squared test^a^.Values are presented as the mean ± SD,One-sample t test ^b^.Score:(I)satisfactory;(II)indeterminate;(III)not satisfactory. PSSI: patient sedation satisfactionassessment tool. Hospitalization days: time for discharge after surgery

The incidence of postoperative adverse effects one day after ERCP in the two groups is shown in Table5. Some patients had nausea, vomiting and abnormal pain in both groups (*p* > 0.05).A small number of patients had fever, and only one patient had polypnea and dyspnoeaon Day 1 after the operation in both groups ( *p* > 0.05).The symptoms of 2 patients were improved by nasal oxygen supplementation. None of the patients reported any experience of pruritus, aspiration pneumoniaor cough.

**Table 5:** Adverse eventsone day after ERCP

	GroupPF(*N* = 199)	GroupPN(*N* = 201)	*P* value	95% CI	Odds ratio
Total adverse events(n)% ^a^	49(23.62%)	52(25.88%)	0.38	0.77–1.22	0.94
Nausea(n)% ^a^	20 (10.05%)	18 (8.96%)	0.35	0.78–1.47	1.14
Vomiting(n)% ^a^	11 (5.53%)	11 (5.47%)	0.49	0.65–1.55	1.01
Pruritus(n)%	0	0	NS		
Aspiration pneumonia(n)%	0	0	NS		
Cough(n)%	0	0	NS		
Polypnea and dyspnoea(n)% ^a^	1 (0.50%)	1 (0.50%)	0.50	0.25–4.03	1.01
Abdominal pain(n)% ^a^	15 (7.54%)	16 (7.96%)	0.44	0.67–1.42	0.94
VAS score(points) ^b^	0.16 ± 0.75	0.13 ± 0.62	0.11	-0.03–0.15	––-
Fever(n)% ^a^	2 (1.01%)	6 (2.99%)	0.08	0.15–1.66	0.33
Medical treatment for adverse events(n)% ^a^	17 (8.54%)	18 (8.96%)	0.44	0.8–1.39	0.95

Values are presented as the frequency (%),Chi-squared test^a^.Values are presented as the mean ± SD. One-sample t test ^b^.*VAS score* visual analogue score, *NA* not available

## Discussion

Our findings show that propofol-nalbuphine sedation for ERCP significantly decreased the incidence of respiratory depression and surgical interruption compared to that with propofol-fentanyl sedation. Moreover, we observed no differences in the analgesic efficacy and haemodynamic features between the two groups.

ERCP is a complex process that requires deep sedation to be completed successfully [21, 22]. However, adverse events, especially respiratory depression, often occur in prone positions during deep sedation [23, 24].Candan Hayturalet al.showed that propofol combined with opioids provides more effective and reliable sedation for ERCP;however, it can still cause respiratory depression and even hypoxia [10,11,12].These results are consistent with ours, in which the incidence of respiratory depression was 12.06% in the PF group, and 14 patients developed hypoxia. Even worse, 5 patients developed severe hypoxia, among whom 2 patients had endotracheal intubation. Therefore, the operation had to be interrupted to correct hypoxia. This event may have reduced the satisfaction of the anaesthesiologist and endoscopist, although the patients were unaware of what had happened.However, such conditions were significantly improved in the PN group, and none of the patients underwent endotracheal intubation. Sedation in the PN group greatly lowered the risks of airway management and enhanced the patient safety during the procedure.

The low incidence of respiratory depression in the PN group may be related to the pharmacological properties of nalbuphine [25].Nalbuphine hydrochloride produces less respiratory inhibition than opioids at the same analgesic dose.It also has a ceiling effect so that respiratory depression does not increase with the dose when it is greater than 30 mg [25,26,27,28]. B. Lefevreet al suggested that nalbuphine should be considered a suitable alternative to fentanyl for use in patients undergoing oral surgery because of less respiratory depression [17].Furthermore, Chaoyi Deng et al. showed that nalbuphine may be a reasonable alternative to sufentanil in patients undergoing colonoscopy^29^.In addition,while nalbuphine can effectively antagonize opioid-induced respiratory depression without adverse endocrine and circulatory changes, nalbuphine still retains its analgesic property [30].In our study,respiratory depression was not associated with age.Patients in both age groups(18–64 y and > 65 y) had more respiratory depression in the PF group than in the PN group. This suggests that there may be no association of age and reduced incidence of respiratory depression with nalbuphine use, which will need further research.

When respiratory depression occurs during ERCP,it may cause hypoxia, which can mostly be treated with the jaw-thrust manoeuvre [29, 31]. This phenomenon was particularly evident in the PN group. Compared to that in the PN group, the hypoxia in the PF group was more severe,as half of the patients were treated with more than one single method (jaw-thrust manoeuvre) to correct the hypoxia. This interrupted the operation, reduced the satisfaction ofthe endoscopists and anaesthesiologists, and increased the difficulties of anaesthesiologists’ work. Therefore, nalbuphine had more advantages over fentanyl in reducing hypoxia in ERCP. However, general anaesthesia with endotracheal intubation became necessary when patients were at high risk for sedation-related adverse events. Compared to propofol-based monitored anaesthesia care, nalbuphine use did not impact the duration of the operation, the outcome of the procedure or patient recovery [32].

Our results are consistent with the findings of others that patients prefer to undergo the ERCP procedure under deep sedation and appropriate analgesia [21, 24].Nalbuphine is a potentanalgesic agent that is similar to morphine [27, 28]. Studies have shown that nalbuphineis widely used in pain management during the perioperative period [29, 33,34,35]. Our results showed that nalbuphine was as effective as fentanyl in reducing pain-induced patient movement, and there was no difference in dosage.Patients in both groups had stable haemodynamics during the operation, which suggested that nalbuphine was effective in relieving painful stimuli and reducing adverse events upon completion of the procedure.

We also compared adverse events, such as nausea, vomiting,pruritus, and pain, after surgery between the two groups.Both groups had a slightly higher incidence of adverse reactions(e.g., nausea and fever)compared to groups receiving analgesic drugs in other studies [36, 37], likely due to procedure (ERCP)-related responses [1].The incidence of vomiting was similar to that in other studies [36, 37].One patient in each group had brief polypnea and dyspnoea after the operation, and both cases improved by nasal oxygen supplementation. Although the reason is still unclear, it may be related to patient anxiety or delayed response of respiratory depression to analgesic drugs [37].Some patients had abdominal pain one day after surgery, but none had severe pain. Few patients received medical treatment for adverse events, and all patients were discharged from the hospital approximately 7 days after the operation. There was no difference in the length of hospitalization between the two groups.Moreover, other researchers have shown that nalbuphine can be used to treat opioid-induced urinary retention and pruritus [38, 39], which makes it an option to be used together with opioids for procedures such as ERCP.

Our study also has some limitations. This is a single-centre trial with a relatively small patient number. A larger-scale study with more patients in multiple centres is needed in the future.

## Conclusions

In conclusion, we demonstrated that sedation with propofol-nalbuphine had advantages over propofol-fentanyl for the ERCP procedure because the former produced less respiratory depression and surgical interruption. Furthermore, nalbuphine can produce adequate analgesia and help to maintain stable haemodynamics in patients undergoing ERCP.Therefore, acombination of propofol and nalbuphineis more efficient and safer for patients during procedural sedation in ERCP.

## Data Availability

All data generated or analysed during this study are included in this published article.

## Abbreviations

ERCP: Endoscopic retrograde cholangiopancreatography
PN: Propofol-nalbuphine
PF: Propofol-fentanyl
MRCP: Magnetic resonance cholangiopancreatography
EUS: Endoscopic ultrasound
PLA: Chinese People's Liberation Army
BMI: Body mass index
ASA: American Society of Anesthesiologists
BIS: Bispectral index
HR: Heart rate
NBP: Noninvasive mean blood pressure
RR: Respiratory rate
SpO2: Oxygen saturation
PSSI: Patient sedation satisfaction assessment tool
VAS: Visual analogue score
